# Prenatal Maternal Antibiotics Treatment Alters the Gut Microbiota and Immune Function of Post-Weaned Prepubescent Offspring

**DOI:** 10.3390/ijms232112879

**Published:** 2022-10-25

**Authors:** Abdullah M. Madany, Heather K. Hughes, Paul Ashwood

**Affiliations:** 1 Department of Psychiatry and Behavioral Sciences, University of California at Davis, 2230 Stockton Blvd., Sacramento, CA 95817, USA; 2 The M.I.N.D. Institute, University of California at Davis, 2825 50th Street, Sacramento, CA 95817, USA; 3 Department of Medical Microbiology and Immunology, University of California at Davis, 3146 One Shields Avenue, Davis, CA 95616, USA

**Keywords:** gut microbiota, antibiotics (ABX), prepubescent, dysbiosis, autism spectrum disorder (ASD), neurodevelopmental disorders (NDD), schizophrenia, weaning, lipopolysaccharide (LPS), *Firmicutes*, *Bacteroidetes*, *Lactobacillus*, *Muribaculaceae*, metabolic pathways, interleukin, cytokines

## Abstract

This study aimed to investigate the immediate and continual perturbation to the gut microbiota of offspring in the weeks post-weaning and how these may be modulated by treating pregnant C57BL/6J dams with antibiotics (ABX). We used a broad-spectrum antibiotic cocktail consisting of ampicillin 1 mg/mL, neomycin 1 mg/mL, and vancomycin 0.5 mg/mL, or vancomycin 0.5 mg/mL alone, administered ad-lib orally to dams via drinking water during gestation and stopped after delivery. We analyzed the gut microbiota of offspring, cytokine profiles in circulation, and the brain to determine if there was evidence of a gut-immune-brain connection. Computationally predicted metabolic pathways were calculated from 16s rRNA sequencing data. ABX treatment can negatively affect the gut microbiota, including reduced diversity, altered metabolic activity, and immune function. We show that the maternal ABX-treatment continues to alter the offspring’s gut microbiota diversity, composition, and metabolic pathways after weaning, with the most significant differences evident in 5-week-olds as opposed to 4-week-olds. Lower levels of chemokines and inflammatory cytokines, such as interleukin (IL)-1α and IL-2, are also seen in the periphery and brains of offspring, respectively. In conclusion, this study shows maternal antibiotic administration alters gut microbiome profiles in offspring, which undergoes a continuous transformation, from week to week, at an early age after weaning.

## 1. Introduction

When major viral pandemics occur, such as SARS, swine flu, MERS, Ebola, Zika, and now COVID-19, there is an increase in prescribing antibiotics (ABX) for use as therapeutic and prophylactic treatments for bacterial coinfection or secondary infection [1], which leads to antibiotic overuse and misuse [2,3,4]. These ABX-treatment courses often coincided with the perinatal period [5,6,7,8,9,10]. Though many studies show the intended benefit to the mother short-term, very little is known about the long-term consequence on the child’s health. The maternal prenatal factors, the delivery method, and the postnatal environment all play a critical role in establishing and maintaining the offspring microbiome [11,12,13]. The gut microbiota comprises various microorganisms, bacteria, viruses, and fungi, that live in the gastrointestinal (GI) tract and play essential roles in health and disease [14]. The bacterial populations are vital for metabolic and immune homeostasis, shaping offspring nutrient processing and disease-fighting capability [15,16,17]. Antibiotics are routinely prescribed perinatally to treat genital tract infections such as Group B *Streptococcus* (GBS), yeast infection, bacterial vaginosis and other sexually transmitted diseases, and urinary tract infections [18,19,20]. Pregnant women are more susceptible to infections, which are dangerous to them and their children and can lead to endometritis, sepsis, and meningitis [21,22]. Therefore, viral outbreaks add to the already high demand for antibiotics during pregnancy. Despite this, there is no solid grasp on the effect on the newborn infant.

Treatment with broad-spectrum antibiotics, such as penicillin plus gentamicin, is considered standard for maternal infections [23]. These antibiotics are considered safe during pregnancy, even though they can potentially cross the placental barrier. In contrast, vancomycin is often given as an alternative to pregnant women with a high risk of penicillin allergy or those infected with multidrug-resistant bacterial strains, as it is poorly absorbed when taken orally therefore, it is given intravenously [24]. Humans begin acquiring their gut microbiota at birth, though prenatal environmental cues can prime the outcomes. The use of antibiotics during delivery has increased in recent years mainly due to current public policies on their use during labor, which was initially instituted to prevent preterm birth, infant mortality, and neurological dysfunction [25] as well as GBS [21,22,26]. Prenatal antibiotic exposure not only affects the mother but is the leading cause of newborn infection. Specific gut microbes in newborns are more prevalent when women give birth vaginally compared to cesarean section, indicating that bacterial colonization was established at birth [27]. In addition, antibiotics have been used as prophylaxis during caesarian birth as those mothers undergoing surgery are more likely to develop infections [28,29]. Although maternal antibiotic use has benefits for treating and preventing infections during pregnancy and delivery [8], little is known about how the gut microbiota and immune system development in their children. 

The gut microbiota directly influences the maturation of the immune system, where deviations from the normal development of the gut microbiota alter outcomes of immune development and potentially increase susceptibility in offspring to various diseases later in life [30,31]. As the gut microbiome plays a critical role in the proper development of the immune systems early in life [32], it becomes ever more crucial to understand how maternal infection, as well as their prevention and treatment measures, may influence this. Maternal infections during pregnancy, such as influenza, are associated with a considerably higher risk of neurologic morbidity in offspring, such as autism spectrum disorders (ASD) and schizophrenia [33,34,35]. ASD comprises a group of heterogeneous neurodevelopmental disorders involving behavioral symptoms, while schizophrenia is a spectrum of conditions involving psychotic symptoms. There is also an increase in inflammatory cytokines and immune cell responses in individuals with ASD [36,37] and schizophrenia [38,39]. At the same time, GI symptoms have been reported in over half of all individuals with ASD [40] and schizophrenia patients on anti-psychiatric medications [41,42]. Although the development of neurological disorders has been linked to infections during pregnancy, less is known of the influence of gut microbiota. In addition to the human cohort studies suggesting that early life exposure to maternal infections can alter the gut microbiota and increase the risk of developing ASD and schizophrenia during the child’s life, animal models of maternal immune activation (MIA) recapitulate features of behavioral and neuronal dysfunction [33,35,43,44,45,46,47,48]. The influence of MIA neuronal remodeling during the prenatal and postnatal developmental stages and the effects on offspring’s immune function and behavioral patterns have been well studied in rodent and non-human primates [43,47,49]. So far, studies have not begun to separate the effects of the antibiotic themselves from the impact of the specific infection they were to treat or determine the antibiotic’s role [50]. The antibiotic treatments strongly disrupt the prenatal microbial communities in the gut [51,52,53]. Studies into the relationship between antibiotic use perinatally and neurological dysfunction have reported that prenatal antibiotics increase ASD risk [54,55]. A better understanding of how the altered gut microbiome influences both health and disease risks in these children is crucial [56,57]. Thus, early infections and immune activation as a result of antibiotic use or a combination of immune activation following antibiotic use could lead to microbial and neurodevelopmental changes.

A considerable number of studies regarding maternal antibiotic treatment during prenatal and early postnatal periods have shown changes in the gut microbiota and altered immune profiles, increasing the risk of disease and morbidity over time [58]. Our study first developed a murine model of exposing pregnant mice to two different antibiotic regimens, an antibiotic (ABX)-cocktail consisting of (ampicillin, neomycin, and vancomycin), versus vancomycin treatment alone, which are not only commonly prescribed to pregnant women but also the newborn [5]. A previous assessment of the infancy stage showed antibiotics caused gut microbiota dysbiosis and immune dysfunction in 3-week-old mice [59]. However, these pre-weaned mice still receive maternal factors. In contrast, our protocol calls for mice to be weaned at the end of 3-weeks-old at postnatal day (p) 21. Only a limited number of studies have been performed during this post-weaning stage. This investigation aimed to determine if prenatal maternal antibiotics treatment affected early post-weaning development in mice, i.e., 4-week-olds and 5-week-olds. This early post-weaning period is often referred to as early adolescence or prepubescence, typically spanning p21–34 [60,61,62]. During post-weaning, there are changes in diet that affect the gut microbiota and often initiate post-weaning dysbiosis. Besides being the beginning of the development of food preferences, this is the period of continued neuronal development. Studies have revealed a significant surge in connections in the brain that is only during the prepubescent period [63]. In this study, we characterize prepubescent gut microbiome and immune dysfunction in offspring following an acute maternal antibiotic treatment late in gestation. We evaluate the early adolescent stage of post-weaning at the end of weeks 4 (p28) and weeks 5 (p35) to determine if there were any continued early life development changes in the absence of direct maternal factors.

## 2. Results 

### 2.1. Maternal ABX-Treatment Continues to Alter the Diversity of the Gut Microbiota in Prepubescent Offspring 

**Figure 1:**
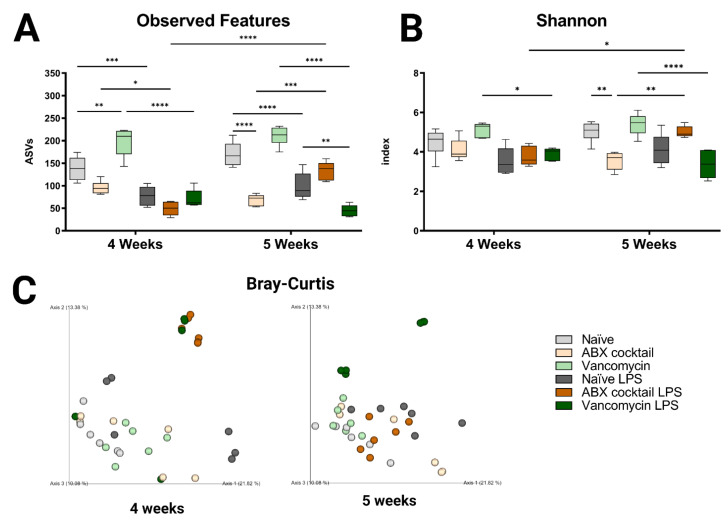
Prenatal ABX administration alters the gut microbiota diversity. (**A**,**B**) α-diversity measured using observed features and Shannon index (**C**) β-diversity indexes measured using principal coordinate analysis (PCoA) plot of Bray–Curtis dissimilarity between samples. Naïve = light grey, ABX cocktail = light brown, Vancomycin = light green, Naïve LPS = grey, ABX cocktail LPS = brown and Vancomycin LPS = green. *n* = 6 for all groups. Two-way ANOVA followed by Tukey post-correction test for multiple comparisons where values represent means ± SEM, * *p* ≤ 0.05, ** *p* ≤ 0.01, *** *p* ≤ 0.001 and **** *p* ≤ 0.0001.

In this study, we investigated the impact of maternal ABX-treatment on the gut microbiota of prepubescent mice offspring, at weeks 4 and 5, corresponding to the prepubescent stage. Lipopolysaccharide, a cell wall constituent of Gram-negative bacteria that is abundant in the GI tract, was injected intraperitoneally to induce a systemic immune response in prepubescent mice, to assess the influence of prenatal ABX treatment on immune responses. The maternal ABX-treatment had no significant effects on body weight group (Figure S1B) when comparing offspring to the naïve controls. However, the cecum in the ABX-cocktail group were larger in the 5-week-olds (*p* ≤ 0.0001) compared to the naïve group (Figure S1C). After LPS challenge, cecums were significantly smaller in the ABX-cocktail group compared to naïve controls (*p* ≤ 0.0001) The size of the cecum in the vancomycin group did not differ from controls. The gut microbiota was analyzed by Illumina 16S rRNA based sequencing of bacterial DNA extracted from fecal pellets. Bacterial taxonomy was used for classification, where the abundance of the amplicon sequence variable (ASVs), also referred to as “observed features”, were determined. The maternal ABX-cocktail treatment led to a decrease in observed features (mean ASVs of 138 and 170 in naïve controls, versus 96 and 69 in the ABX-cocktail group, two-way ANOVA, *p* = 0.0573 and *p* ≤ 0.0001, at 4 and 5 weeks, respectively) (Figure 1A). In contrast, the vancomycin group had an increase in observed features (mean ASVs of 198 and 211 in the vancomycin group, *p* = 0.0011 and *p* = 0.0909), at 4 and 5 weeks, respectively. Following LPS challenge, differences in the observed features in all groups were seen, with the most significant differences occurring in the vancomycin groups (*p* ≤ 0.0001). When the vancomycin group was challenged with LPS, observed features were decreased significantly at 5 weeks (mean ASVs of 99 to 45, *p* = 0.0046, at 5 weeks) compared to the naïve offspring challenged with LPS. When challenged with LPS, there was an increase in observed features in the ABX-cocktail (mean ASVs of 69 to 134, *p* = 0.0002, at 5 weeks). When comparing the 4-week-olds to the 5-week-olds, after LPS challenge in the ABX-cocktail treatment group, observed features were significantly different (mean ASVs of 49 versus 134, *p* ≤ 0.0001). 

To better understand the impact on α-diversity, another metric measuring species evenness was used, i.e., the Shannon index. Here, the maternal ABX-cocktail treatment also led to a decrease in α-diversity (mean Shannon index 4.5 and 5.0 in naïve controls, versus 4.1 and 3.6 in the ABX-cocktail group, *p* = 0.9948 and *p* = 0.0019, at 4 and 5 weeks, respectively) (Figure 1B). The vancomycin alone group did not have significant differences compared to the naïve controls. However, a significant decrease in Shannon index was seen in the vancomycin group after the LPS challenge (mean Shannon index 5.2 and 5.4 in the vancomycin group, versus 3.9 and 3.4 in the vancomycin LPS group, *p* = 0.0212 and *p* ≤ 0.0001, at 4 and 5 weeks, respectively). Finally, when comparing the 4-week-olds to the 5-week-olds, after the LPS challenge, there was a significant increase in diversity in the ABX-cocktail group (mean Shannon index of 3.8 versus 5.0, *p* = 0.0129). Next, to determine the impact on β diversity, cluster analysis with Bray–Curtis was performed to assess dissimilarity between groups. Maternal ABX-treatment led to a clear shift in β-diversity as seen by the analysis of community structures. The ABX-cocktail group clusters further away from the naïve controls at baseline but are clustered more closely after the LPS challenge (Figure 1C). In general, the LPS challenge led to tighter clusters than in unchallenged conditions/baseline. Alterations in species richness and evenness was seen in the offspring of the ABX-treated groups, with an increased dissimilarity in both 4-week-old and 5-week-old prepubescent mice. In summary, these results indicate that the offspring’s gut microbiota is disrupted, leading to dysbiosis as a result of maternal ABX-treatments, and that systemic immune response with the LPS challenge exacerbates this dysbiotic phenotype. Therefore, these results highlight that acute maternal antibiotic treatment can cause substantial gut microbiota perturbation in prepubescent offspring, which merits future into preventive health strategies.

### 2.2. Maternal ABX-Treatment Modulates Offspring Gut Microbial Colonization Patterns

**Figure 2:**
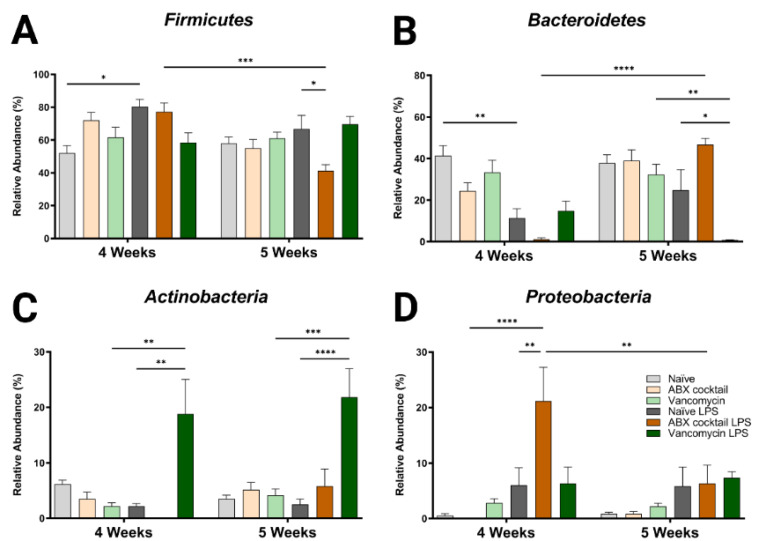
Maternal ABX alters offspring phylum populations. Relative abundances of (**A**) *Firmicutes*, (**B**) *Bacteroidetes*, (**C**) *Actinobacteria,* and (**D**) *Proteobacteria* at 4 and 5 weeks. Naïve = light grey, ABX cocktail = light brown, Vancomycin = light green, Naïve LPS = grey, ABX cocktail LPS = brown and Vancomycin LPS = green. *n* = 6 for all groups. Two-way ANOVA followed by Tukey post-correction test for multiple comparisons where values represent means ± SEM, * *p* ≤ 0.05, ** *p* ≤ 0.01, *** *p* ≤ 0.001 and **** *p* ≤ 0.0001.

To further characterize the dysbiotic phenotype of the prepubescent offspring of ABX-treated dams, we examined how the altered α/β diversity contributes to the taxonomic community structure. Only four bacteria phyla were found in all groups; *Firmicutes* and *Bacteroidetes* were the dominant phyla accounting for most of the assigned sequences, while *Proteobacteria* and *Actinobacteria* had relatively low abundances (Table S1). The maternal ABX-cocktail treatment led to an increase in *Firmicutes* and a decrease in *Bacteroidetes* but did not reach significance (mean relative abundance from 52% to 72% in the *Firmicutes*, and from 41% to 25% in the *Bacteroidetes*) in the 4-week-olds as compared to the naïve controls (Figure 2A,B). Changes have previously been reported following in vivo LPS challenge at the phylum level [64,65]. In this study, we observed alterations in the microbiota in offspring, with increased *Firmicutes* and decreased *Bacteroidetes* seen in naïve control mice at 4-week-olds after LPS challenge (*p* = 0.0132 and *p* = 0.0029). These changes were less pronounced at 5 weeks. In the ABX treatment group, there was a decrease in *Firmicutes* and increased *Bacteroidetes* at 5 weeks compared to the controls (mean relative abundance from 67% to 41% in the *Firmicutes*, and from 41% to 25% in the *Bacteroidetes*, *p* = 0.0402 and *p* = 0.0910). It has previously been shown that an ABX-induced decrease in diversity resulted in an increase in the *Firmicutes* to *Bacteroidetes* (F/B) ratio [66]. Increased or decreased F/B ratio is a characteristic of dysbiosis. 

When comparing the 4-week-olds to the 5-week-olds, the ABX-cocktail led to a significant difference in *Firmicutes* and *Bacteroidetes* only after LPS (mean relative abundance of 77% versus 50% for the *Firmicutes*, and 1% versus 47% for the *Bacteroidetes*, *p* = 0.0004 and *p* ≤ 0.0001). Additionally, after the LPS challenge, a significant difference was seen in the 5-week-olds from the vancomycin group, which was reduced compared to vancomycin without LPS stimulation (mean relative abundance *Bacteroidetes* 32% versus <1%, *p* = 0.0014 at 5 weeks), and the vancomycin group challenged with LPS was lower than the naïve controls challenged with LPS (mean relative abundance *Bacteroidetes* 25% versus <1% *p* = 0.0405 at 5 weeks). 

*Actinobacteria* and *Proteobacteria* populations were less abundant, resulting in less variation between groups and no significant differences at either age when comparing the naive controls to the ABX-cocktail and vancomycin groups at baseline. However, after the LPS challenge, the 4-week-olds did show significant differences from their baseline (mean relative abundance from 2% to 19% in the *Actinobacteria*, and from 0% to 21% in the *Proteobacteria*, *p* = 0.0011 and *p* ≤ 0.0001, at 4 weeks) and after LPS challenge in the group (mean relative abundance from 2% to 19% in the *Actinobacteria*, and from 6% to 21% in the *Proteobacteria*, *p* = 0.0011 and *p* = 0.0059, at 4 weeks) (Figure 2C,D). At 5-week-olds, *Actinobacteria* was also significantly increased in the vancomycin group following the LPS challenge (mean relative abundance from 4% to 22% in the *Actinobacteria*, *p* = 0.0002, at 5 weeks). In the ABX-cocktail group there was a difference after the LPS challenge at the two time points in the *Proteobacteria* (mean relative abundance from 21% at 4 weeks to 6% at 5 weeks, *p* = 0.0079).

**Figure 3:**
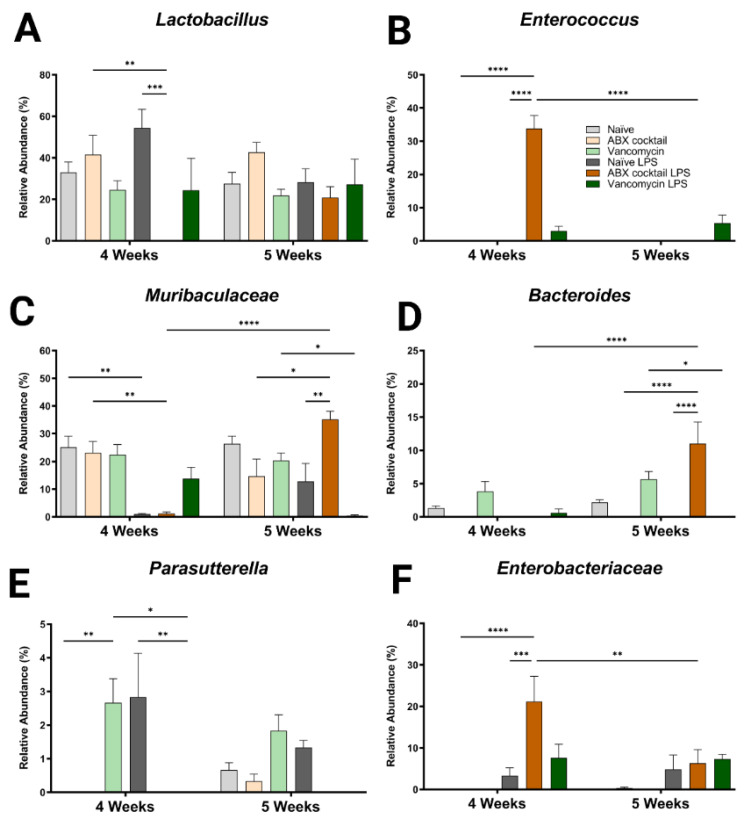
Maternal ABX alters offspring genus populations. Relative abundances of (**A**) *Lactobacillus*, (**B**) *Enterococcus*, (**C**) *Muribaculaceae*, (**D**) *Bacteroides*, (**E**) *Parasutterella*, and (**F**) *Enterobacteriaceae* at 4 and 5 weeks. Naïve = light grey, ABX cocktail = light brown, Vancomycin = light green, Naïve LPS = grey, ABX cocktail LPS = brown and Vancomycin LPS = green. *n* = 6 for all groups. Two-way ANOVA followed by Tukey post-correction test for multiple comparisons where values represent means ± SEM, * *p* ≤ 0.05, ** *p* ≤ 0.01, *** *p* ≤ 0.001 and **** *p* ≤ 0.0001.

In addition to the altered phyla, maternal ABX-treatment alters the colonization of gut microbiota, ranging from large expansions to complete loss of populations at lower levels of categorization. Therefore, we next assessed the generas from 12 most abundant sequence reads, which happen to account for most of the total reads. Here, some of the dominant genera were similar compared to the controls at baseline (*Lactobacillus* spp. and *Muribaculaceae* family), while other genera appeared to colonize more or less abundantly (*Bacteroides* and *Parasutterella* spp.), or only occurred after the LPS challenge (*Enterococcus* and *Enterobacteriaceae* spp.) (Figure 3). Regardless, there were significant differences in the responses to the LPS challenge, mainly in the ABX-cocktail group. *Lactobacillus* spp. belongs to the phyla *Firmicutes* and is the most abundant genus in prepubescent mice. The *Lactobacillus* spp. populations in ABX-cocktail group were entirely gone after the LPS challenge (mean relative abundance from 0% versus 42% in the ABX-cocktail, and 0% versus 54% in the naïve LPS, *p* = 0.0099 and *p* = 0.0002, at 4 weeks) (Figure 3A). No differences were seen between the vancomycin group and the naïve controls either with or without LPS challenge. *Enterococcus,* also from phyla *Firmicutes*, was not expressed at baseline; however, this population significantly increased after the LPS challenge in the ABX-cocktail group only at 4 weeks (mean relative abundance of 34% versus 0% in the ABX-cocktail vs. naïve controls after LPS *p* ≤ 0.0001), but was absent at 5 weeks (34% versus 0%, and *p* ≤ 0.0001) (Figure 3B).

*Muribaculaceae* family is the most abundant of the phyla *Bacteroidetes*. The populations of *Muribaculaceae* family in both naïve controls and ABX-cocktail group were decreased following the LPS challenge in the 4-week-old (mean relative abundance of 0% from 23% in the ABX-cocktail, *p* = 0.0044) (Figure 3C). At 5 weeks, after the LPS challenge, *Muribaculaceae* family were increased in the ABX group compared to no LPS challenge and to naïve mice following LP challenge (mean relative abundance of 35% from 15% in the ABX-cocktail, and 35% from 12% in the naïve LPS, *p* = 0.0107 and *p* = 0.0035). *Muribaculaceae* family in the vancomycin following LPS group decreased at 5 weeks (mean relative abundance of 1% from 20%, *p* = 0.0158). *Bacteroides* spp. also from the phyla *Bacteroidetes,* were depleted entirely at baseline in the ABX-cocktail group at 4 and 5 weeks; however, after LPS challenge in the ABX group at 5 weeks *Bacteroides* spp. was significantly increased compared to ABX without LPS challenge (*p* ≤ 0.0001) (Figure 3D). 

*Parasutterella* spp. belongs to the phyla *Proteobacteria* and was only seen in the vancomycin group at baseline at 4 weeks (*p* = 0.0139) and also populated in controls following the LPS challenge while the vancomycin group had the opposite effect (*p* = 0.0078), compared with naïve controls (Figure 3E). Though the naïve controls and the ABX-cocktail as well as the vancomycin group have populations of *Parasutterella* spp. at 5 weeks the naïve control are the only group that maintains this population following the LPS challenge. *Enterobacteriaceae* spp. belong to the phyla *Proteobacteria*. This population significantly increased after the LPS challenge in the ABX-cocktail LPS group only (compared with the ABX-cocktail without challenge (*p* = 0.0001) and compared with the naïve group following the LPS challenge (*p* ≤ 0.0002), at 4 weeks (Figure 3G). Comparing the relative abundance of specific bacteria across taxonomic levels is essential for determining how maternal antibiotic affects prepubescent offspring gut microbiota bacterial colonization. The remaining genres also show significant differences (Figure S2). In summary, although vancomycin alone and ABX-treatment regimens alter the gut microbiota, the most robust changes happened in response to the ABX-cocktail treatment in the 5-week-old mice.

### 2.3. Maternal ABX-Treatment Results in Alteration of Bacterial Metabolic Pathways in Offspring after Weaning

**Figure 4:**
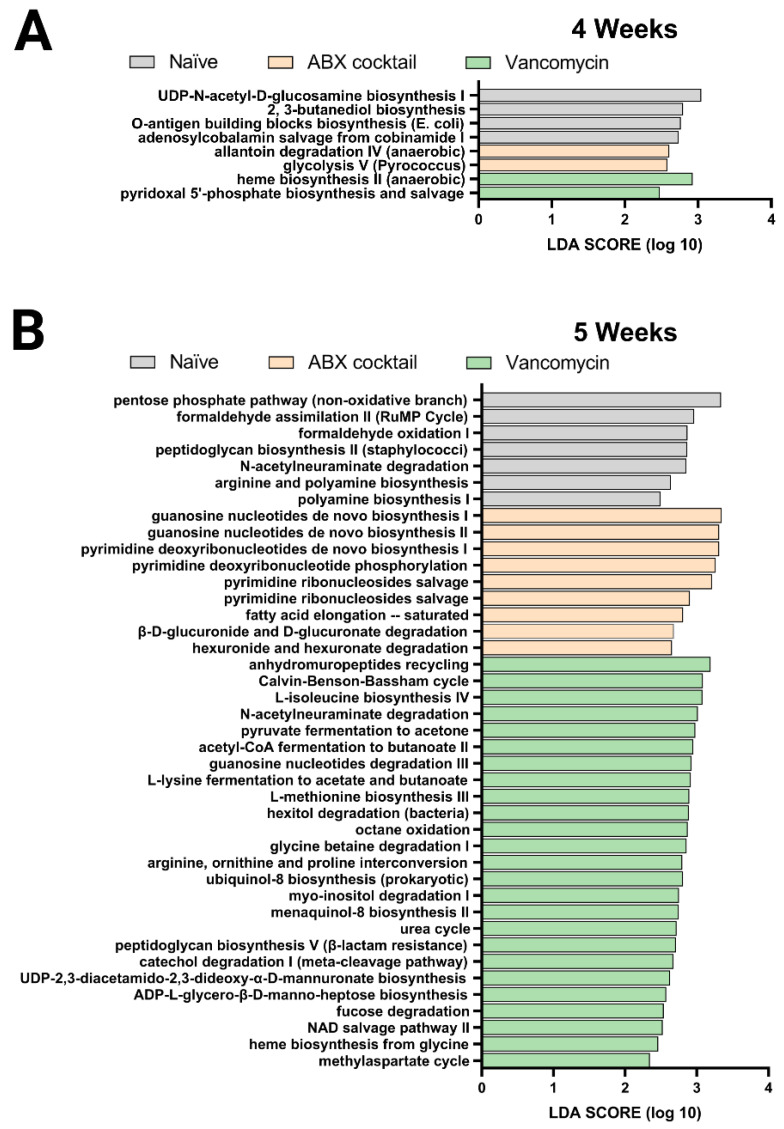
PICRUSt predictions of metabolic pathways. Functional summary for MetaCyc pathways, Linear discriminant analysis (LDA) effect size (LEfSe) analysis revealed significant bacterial differences in gut microbiota between the antibiotic-treated groups and naïve group. from (**A**) 4 weeks and (**B**) 5 weeks. LDA scores (log10) > 2 and *p* < 0.05 are shown. Naïve = light grey, ABX cocktail = light brown, Vancomycin = light green, with *n* = 12 per group.

Finally, we performed the PICRUSt analysis to predict the relative abundance of metabolic pathways in the offspring gut microbiota altered by maternal antibiotic treatment. Here, the previous 16s rRNA sequenced data was used to infer the functional metabolic capability of the gut microbiota of 4 and 5-week-old mice. The functional capacities were ranked according to effect size between the three groups: the naïve, ABX-cocktail, and vancomycin groups with LPS challenges serving as a subgroup. This allowed for the identification of discriminatory pathways purely based on maternal ABX-treatment. The linear discriminative analysis (LDA) effect size (LEfSe) analysis of MetaCyc pathways was used to determine where several metabolic pathways were biologically significantly altered, with the majority coming from the vancomycin groups in the 5-week-olds (Figure 4).

The ABX-cocktail group had significant differences in pathways associated with glycolysis V and allantoin degradation IV. In contrast, the microbiota in the vancomycin group had differences in pyridoxal 5’-phosphate and heme biosynthesis in the 4-week-olds (Figure 4A). The gut microbiota of 5-weeks-olds from the ABX-cocktail group had predicted increases in pathways associated with guanosine biosynthesis, pyrimidine phosphorylation biosynthesis, fatty acid elongation–saturated (Figure 4B), while in the vancomycin group, there were increases in a variety of pathways, including methylaspartate cycle, heme biosynthesis, NAD salvage pathway II, fucose degradation, ADP-L-glycero-β-D-manno-heptose biosynthesis, UDP-2,3-diacetamido-2,3-dideoxy-α-D-mannuronate biosynthesis, catechol degradation I, peptidoglycan biosynthesis V, urea cycle, ubiquinol-8 biosynthesis, glycine betaine degradation I, octane oxidation, hexitol degradation, L-methionine biosynthesis III, L-lysine fermentation to acetate and butanoate, guanosine nucleotides degradation III, acetyl-CoA fermentation to butanoate II, pyruvate fermentation to acetone, L-isoleucine biosynthesis IV, Calvin-Benson-Bassham cycle. (Figure 4B). 

In summary, The LDA effective size (LEfSe) was performed to identify MetaCyc pathways with statistically differential abundance between the ABX-cocktail and vancomycin group, where the 5-week-olds from the vancomycin group had carbohydrate biosynthesis, amino acid biosynthesis, cofactor, carrier, and vitamin biosynthesis, secondary metabolites biosynthesis, and other biosynthesis pathways with LDA score greater than 2.0. In contrast, the ABX-cocktail only had nucleotide and nucleoside biosynthesis and fatty acid and lipid biosynthesis pathways with LDA score greater than 2.0. The effect of maternal ABX-treatment on metabolism-related pathways were more abundant in the vancomycin group than in the ABX-cocktail group. However, based on these predicted pathways, there are alterations in 4-weeks-olds, though to a lesser extent, we see that antibiotics significantly alter essential metabolic pathways in the prepubescent stage. Combined with altered diversity and taxonomic population shifts, these findings paint a picture of gut microbiota dysbiosis [67]. Therefore, overall, our 16s rRNA sequence data suggest that the maternal antibiotics treatment results in dysbiotic post-weaned prepubescent offspring with increasing significance later in the prepubescent period, suggesting that the key developmental process that occurs within might be hindered due to the altered metabolic potential of the gut microbiota.

### 2.4. The Maternal ABX-Treatment Influences Cytokine Levels in the Prepubescent Offspring

**Figure 5:**
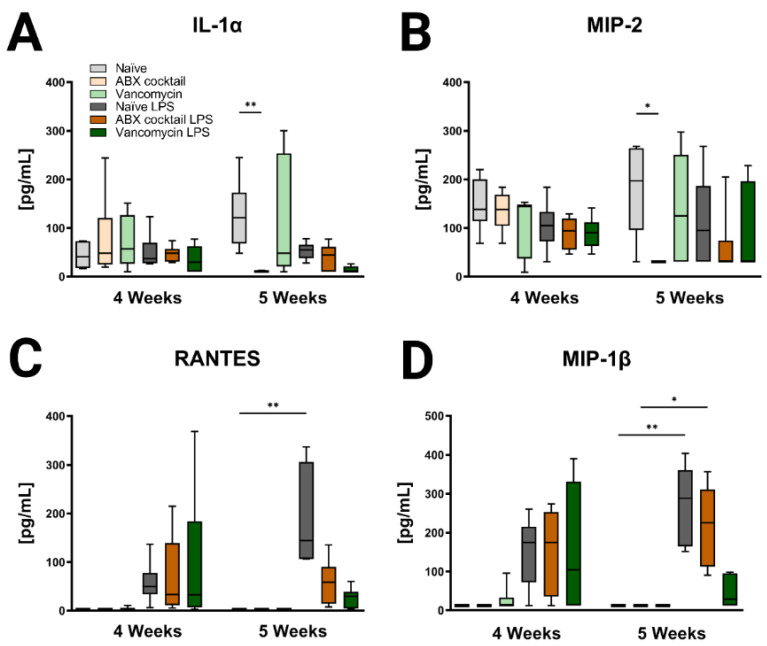
Inducible serum cytokines and chemokines are affected by maternal ABX treatment. The data represents the responses of (**A**) IL-1α, (**B**) MIP-2, also known as CXCL2, (**C**) RANTES, also known as CCL5, and (**D**) MIP-1 β, also known as CCL4 at 4 and 5 weeks. Naïve = light grey, ABX cocktail = light brown, Vancomycin = light green, Naïve LPS = grey, ABX cocktail LPS = brown and Vancomycin LPS = green. *n* = 6 for all groups. Kruskal–Wallis with Dunn’s multiple comparison test. Values represent median + interquartile range means ± SEM, * *p* ≤ 0.05, ** *p* ≤ 0.01.

**Figure 6:**
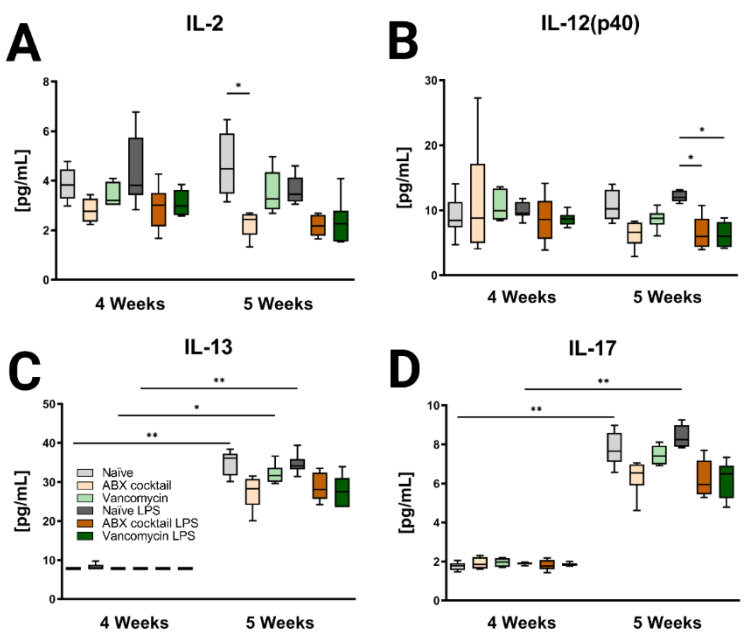
Inducible brain cytokines are affected by maternal ABX treatment. Data showing the responses of (**A**) IL-2, (**B**) IL-12(p40), (**C**) IL-13, and (**D**) IL-17 at 4 and 5 weeks. Naïve = light grey, ABX cocktail = light brown, Vancomycin = light green, Naïve LPS = grey, ABX cocktail LPS = brown and Vancomycin LPS = green. *n* = 6 for all groups. Kruskal–Wallis with Dunn’s multiple comparison test. Values represent median + interquartile range means ± SEM, * *p* ≤ 0.05, ** *p* ≤ 0.01.

Lastly, immune profiles were assessed in the prepubescent offspring to see if there were differences associated with maternal ABX-treatment. Cytokines in the periphery and brain were altered following antibiotic treatment regimens (Figure 5, Figure 6, Figure S3 and Figure S4). In the 5 weeks olds, Maternal ABX-cocktail led to a significant decrease in IL-1α and macrophage inflammatory protein-2 (MIP-2, also known as CXCL2) in the baseline serum (Kruskal–Wallis test, *p* = 0.0012 and *p* = 0.0431), while the vancomycin treatment had no significant effect on either IL-1α and MIP-2, also known as CXCL2 (Figure 5A,B). Following the LPS challenge, cytokines increased overall in serum at both time points though most significance were seen in the 5-week-olds. LPS challenge resulted in increased proinflammatory cytokines with significant increases in Regulated on Activation, Normal T cell Expressed and Secreted (RANTES, also known as CCL5) and MIP-1β, also known as CCL4 in the serum of the naïve control (Kruskal–Wallis test, *p*  =  0.0015 and *p* = 0.0329, respectively). This effect was not seen in the maternal ABX-treatment offspring following the LPS challenge. There were no significant differences seen in the 4-week-olds (Figure 5C,D). 

Brain tissue was then assessed for the CNS immune profile. The ABX-cocktail led to less IL-2 (*p* = 0.0109) at baseline without LPS challenge, and IL-12(p40) following LPS challenge (*p* = 0.0353) in the 5-week-olds (Figure 6A,B). The brains of 5-week-old prepubescent mice produced significantly more IL-13 (*p* = 0.0030), and IL-17 (*p* = 0.0048) than at 4-week-olds (Figure 6C,D), this significant increase was not seen in the offspring of the ABX-treated dams. Additional significant cytokine changes were also seen in the serum and brain (Figures S3 and S4). In summary, the results show that maternal ABX-treatments resulted in an altered immune profile in the periphery as well as the CNS of prepubescent offspring, due to the interaction of the gut microbiota with the immune system. 

## 3. Materials and Methods

### 3.1. Animals and Experimental Design

All dams and sires were mice of the C57BL/6J strain purchased from Jackson Laboratory (Sacramento, CA, USA). The mice were given a week to acclimate to the vivarium before breeding started. The dams were mated between 6–8 weeks of age, with one male and two females per cage. The embryonic (E) day was determined by the presence of a vaginal plug marking E0.5, and the males were then removed from the cage. Dams were either untreated controls (naïve group) or treated orally via drinking water from E14.5 until birth, i.e., acutely (5 days) with either vancomycin (0.5 mg/mL) (Alvogen, Morristown, NJ, USA) alone (vancomycin group), or ampicillin (1 mg/mL), neomycin (1 mg/mL) (MilliporeSigma, Burlington, MA, USA) and vancomycin (0.5 mg/mL) in combination (ABX-cocktail group). This regimen is a modification to is consistent with standard antibiotic administration used in the protocol described by Rakoff-Nahoum et al. [68]. Here, antibiotic treatment did not affect water consumption or chow. Fresh antibiotics were maintained in the drinking water until birth when the dams were then switched back to regular drinking water. After birth, dams were placed in individual cages, with regular drinking water, and there was no cross-fostering. The average litter size was 7–8 pups, and antibiotic treatment did not alter litter size. This study focused on the effects of acute maternal antibiotics during pregnancy, specifically on the offspring’s prepubescent development. The offspring were kept in their groups 2–4 per cage after weaning. This study utilized both male and female offspring. The ages were selected to represent early post-weaning prepubescent development time points in the mice, i.e., from 3 weeks to 4 weeks and from 4 weeks to 5 weeks. Each experimental time point consisted of males and females from each of the dams in the groups, and half the offspring were stimulated with LPS before analysis. The untreated antibiotic naïve group served as the maternal ABX-treatment control, also, the PBS-treated offspring served as LPS controls, i.e., baseline (Figure S1A). The dams, sires, and offspring mice were kept on a standard chow diet throughout the study and housed under specific pathogen-free conditions with a 12/12 h light/dark cycle on the UC Davis Medical Center campus. All animal studies conform to NIH policies, and ethical approval was obtained from the Institutional Animal Care and Use Committee IACUC at University of California Davis under protocol #20808. 

### 3.2. Immune Challenge and Sample Collections

A systemic immune response was initiated by an intraperitoneal (IP) injection with 5 mg/kg of body weight lipopolysaccharide (LPS) from *Escherichia coli* O55:B5 (MilliporeSigma, Burlington, MA, USA) into 4-week-old and 5-week-old prepubescent mice. An equivalent amount of phosphate-buffered saline (PBS) was given to the controls. The fecal samples were collected 24 h post-injections, and animals were euthanized for serum and brain tissue collections. The peak brain immune response after the LPS challenge is seen at this time. Additionally, it also provided time for gut microbiota changes to the immune stimulation. Fecal samples were collected from individually isolated mice that were placed in sterile cages to collect fecal pellets. The collected samples were stored directly on dry ice before being transferred to −80 °C until analysis. Animals were then anesthetized with isoflurane. Blood was drawn into an uncoated capillary tube via a nick in the cardiac artery. The blood was transferred to a tube and allowed to clot at room temperature before placing it on ice. After a 10 min centrifugation at 4 °C and 13,000× *g*, blood serum was collected and stored at −80 °C until analysis. Following blood collection, brains were perfused with HBSS through the left ventricle; then, the extracted brain was hemi-sectioned for protein extraction. The cecum was also removed from the gut for weight measurements. All tissue samples were immediately frozen on dry ice and then stored at −80 °C until analysis.

### 3.3. Microbiota and Cytokine Analysis

For the microbiota analysis, bacterial DNA was extracted from fecal samples of the naïve, ABX-cocktail and vancomycin groups at 4 and 5 weeks. Using barcoded primers, the 16S rRNA amplicon sequencing of the V4 region was performed using the MiSeq platform (Illumina, San Diego, CA, USA). In brief, the Illumina demultiplexing, merging of the reads and trimming of the barcodes and primers, and the QC were performed by the UC Davis sequencing core. FastQ files were then analyzed using the QIIME2 software package v. 2021.2 [69]. DADA2 plugin [70] was used to filter and trim the files reads first then, remove chimeras, and count abundance were then determined. We then used the q2-feature-classifier plugin [71], which was used to identify the most likely original taxonomic sequences in the sample based on the SILVA 138 database [72] and can be represented on bar plots. Finally, we used the q2-emperor plugin, which was used to view sample taxonomic diversity profiles. β-diversity was represented on PCoA plots.

For the cytokine analysis, serum was collected from the clotted blood samples. Brain samples were collected from mice and immediately stored at −80 °C until use. Individual brain samples were suspended in PBS with a protease inhibitor cocktail (Cell Signaling Technology, Danvers, MA, USA), then physically disrupted by vortexing thoroughly, followed by brief sonication. The disrupted pellets were centrifuged (30,000× *g* for 10 min at 4 °C) to precipitate insoluble materials. The hippocampus was processed similarly to the whole brain.

The concentration of protein extracts in the supernatant of the brain were measured by using the BCA protein assay (Thermo Scientific, Waltham, MA, USA). The quantification of cytokines was determined using mouse reactive Milliplex™ multiplexing bead immunoassays (Millipore, Burlington, MA, USA). Which allows for the simulatinuse detection of: G-CSF, GM-CSF, IFN-γ, IL-1α, IL-1β, IL-2, IL-4, IL-5, IL-6, IL-7, IL-9, IL-10, IL-12 (p40), IL-12 (p70), IL-13, IL-15, IL-17, IP-10, KC, MCP-1, MIP-1α, MIP-1β MIP-2, RANTES, and TNF-α. Serum was run at equal volumes, while brain supernatant was run at 3.2 ug/uL in accordance with the instructions of the manufacturer’s protocol. In brief, the supernatant was incubated with antibody-coupled beads. After a series of washes, a biotinylated detection antibody was added to the beads, and the reaction mixture was detected by the addition of streptavidin-conjugated to phycoerythrin. The bead sets were analyzed using a flow-based Luminex™ 100 suspension array system on the Bio-Plex 200 platform (Bio-Rad Laboratories, Hercules, CA, USA). The unknown sample cytokine concentrations were then calculated by Bio-Plex Manager software using a standard curve derived from the known reference cytokine concentrations supplied by the manufacturer.

### 3.4. Computation Analysis and Statistics

The computational analysis was performed to predict metagenome functional content in each sample of the naïve, vancomycin, and ABX-cocktail groups. Briefly, the 16S ASVs were normalized and aligned to a reference phylogenetic tree then the predicted functional gene families and copy numbers for each specific ASV were found. 

We ran the full PICRUSt2 pipeline command in QIIME2. As output, we obtained MetaCyc pathway abundance predictions based on enzyme profile enzyme commission (EC) code and abundances [73]. LEfSe is an algorithm that identifies genomic features characterizing the differences between biological groups. We used this to compare the naïve, vancomycin, and ABX-cocktail groups. We used the LPS treatment as a subclass in this analysis. The grouped data were analyzed using the non-parametric factorial Kruskal–Wallis with a significance set to <0.05 and pairwise Wilcoxon’s tests. Finally, LEfSe uses linear discriminant analysis (LDA) to estimate the effect size of each differentially abundant feature [74]. 

The LDA threshold was set at ± 2. LEfSe data analysis was prepared using the Huttenhower Lab Galaxy server. Statistical analysis of the gut microbiota using two-way ANOVA followed by the Tukey post hoc test for multiple comparisons. This data is presented as mean ± standard error of the mean (SEM). LDA analysis incorporates both Kruskal–Wallis and pairwise Wilcoxon’s tests. The Kruskal–Wallis was used for the analysis of cytokine data, followed by Dunn’s post hoc test for multiple comparisons. The gut microbiome data was analyzed using Qiime2 software, and all data were graphed using GraphPad Prism v 9.0 Software (GraphPad, LA Jolla, CA, USA). The final figures were assembled using Biorender software 2022 (Toronto, ON, CANADA). With all significant differences measured represented by * *p* < 0.05, ** *p* < 0.01, *** *p* < 0.001, and **** *p* < 0.0001. 

## 4. Discussion

It is known that events occurring during the perinatal period have severe consequences for long-term risk for disease or neurodevelopmental disorders like ASD and schizophrenia [31,34,45,47,54,55,75,76,77]. Consequently, numerous studies have focused on the prenatal and early postnatal period before weaning. However, various development processes occur after weaning, and the relative importance of this post-weaning maturation remains less clear. This is a critical time point in humans as a significant shift in the bacterial populations of gut microbiota occurs due to children switching to a solid and more varied diet; these and other age-related changes have huge implications on health [78]. In addition, in social isolation models of initiating neuropsychiatric disorders, the prepubescent period is a key time in behavior development [79,80,81]. The gut microbiota produces a large number of compounds that can influence health; most are beneficial [82], though others can be toxic, and changes in bacterial populations could potentially contribute to an increased risk of neurodevelopmental disorders.

Therefore, this study addressed whether maternal antibiotic treatment during pregnancy evokes lasting changes in the gut microbiota and immunological responses of post-weaned prepubescent mice. We profiled the gut microbiota of the offspring and evaluated their serum and brain immunological cytokine levels in the early post-weaning period. To model this gut microbiota development in mice, we used prepubescent pups that stop drinking mother’s milk and switched to eating chow exclusively which begins at week 4. The weaning of mice comprises both dietary and environmental changes, which influence homeostasis and can lead to diet-induced dysbiosis [83]. As shown by our results, the ABX-cocktail alters the α-diversity, i.e., observed features and Shannon, we see significant decreases, which indicate the diversity within microbial communities [84] was changed in the offspring as a response to maternal ABX treatment. We also saw β-diversity indexes, which measure dissimilarity, being influenced by ABX treatment. We further characterized the gut microbiota profiles by comparing the relative abundance of the gut microbiota bacterial populations using the16s RNA genes [85], where all phyla were affected. The ABX-cocktail gut microbiota bacterial populations were either increased, decreased at baseline, or completely eliminated in response to the LPS as with *Bacteroidetes* or *Actinobacteria* or initiated as with *Proteobacteria* as compared to the naïve controls. Such shifts at the phyla level can be quite detrimental and have profound consequences for gut health.

Though the shifts at the baseline phyla level did not reach significance, there are significant alterations at the genus level at baseline in response to maternal ABX-treatment. For instance, *Bacteroides* spp. are completely missing from the ABX-cocktail group. The *Bacteroides*, has the capacity to produce an array of enzymes that breakdown carbohydrate [86], while *Parasutterella* spp. are a vital component of the human and mouse gut microbiota [87], is only seen in the vancomycin group at 4 weeks. Additionally, the most dominant gut microbiota genus member *Lactobacillus* spp. are wiped out in response to LPS. *Lactobacillus* spp. alter the expression of GABA receptors and influence the production of oxytocin [88]. Changes were also seen in less abundant taxonomic populations that could alter gut homeostasis, for example, the ABX cocktail group had significantly higher *Enterobacteriaceae* and *Enterococcus* spp. *Enterobacteriaceae* spp. are Gram-negative bacteria, some of which are considered commensal. However, some members are pathogenic and have been associated with inflammatory bowel disease [89]. The common *Enterococcus* species typically are non-deleterious in healthy individuals; however, these can cause health problems in individuals with a weakened immune system [90]. When mice are challenged with LPS, their commensal microbe populations are altered and affect the gut microbiota such that microbe populations that are normally either under-represented or potentially opportunistic may grow [91,92,93]. In addition, the LPS challenge has been shown to affect α and β-diversity [65,94]. In general, we observed the exacerbation of the dysbiotic profile in the offspring challenged with LPS. We evaluated vancomycin alone as it does not cross the gut vascular barrier (GVB) when administered orally [95], unlike the other components of the ABX-cocktail that can be absorbed through the GI tract. Interestingly, maternal vancomycin alone was shown to increase diversity at 4–5 weeks, despite many studies showing vancomycin treatment decreased diversity in offspring when given early in life [96,97,98]. The commonly used metagenome prediction tool Picrust enabled the extrapolation of the potential metabolic pathway. Though vancomycin treatment alone leads to fewer diversity differences, we see significantly more pathways altered as compared to the ABX-cocktail. Therefore, a single antibiotic use can result in long-lasting detrimental shifts so the gut microbiome.

Factors from the circulation can ultimately reach the brain after crossing the blood–brain barrier (BBB), many of these could be derived from the gut and thus, there exists a form of cross communication with the GI system [99,100]. In the periphery, we demonstrated that the maternal ABX-cocktail decreases cytokine IL-1α and MIP-2, also known as CXCL2 levels in the serum of mice. Cytokines are important for proper neurodevelopment and may enter the brain and affect function, and thus there is tight regulation of cytokine passage [101]. The cytokines profiles were used as a comparative measure to determine the stability of the immune response of offspring. There is a significant reduction in proinflammatory cytokines in the serum and brain of mice from maternal antibiotic treatment. The cytokines produced are vital mediators of intercellular signaling, communication, and inflammatory responses. Proinflammatory cytokines can also disrupt the gut microbiota and impact gut vascular barrier function [15,16]. 

The results of the current study also indicate that the more significant impairments in gut microbiome development were seen in 5-week-olds. Despite a number of factors changing at week 4, i.e., placed in a new cage (environment) and removed from milk (diet), we see greater alterations happening at week 5. In addition, the 5-week-old mice produce significantly more IL-13 and IL-17 than 4-week-old mice. Reduced proinflammatory cytokines are potentially due to reduced response to LPS challenge and may result in less immune and brain interactions. The extended dysbiotic period from infancy into the prepubescence stage, shown by decrease in bacterial diversity and decreased of key populations, may result in dysfunction in immune response [102]. Recent studies using similar antibiotic treatments show IL-17 cells being affected and suggest IL17-mediated responses are key regulators of early dysbiosis [103].

Our study has several limitations. First, we did not evaluate the maternal gut microbiota to confirm dysbiosis in the dams; we instead focused on the offspring. Based on published studies, the concentrations and timeframes we chose would elicit a dysbiotic response at the end of antibiotic treatment. These studies have demonstrated that maternal antibiotics during pregnancy would disrupt the maternal and offspring gut microbiota. We intentionally focused our study at 4 to 5 weeks and not across the life-span as little is known about this period of development. Additionally, components of the ABX-cocktail have the potential to cross the GVB. Therefore, we cannot rule out any direct impact on the embryos. Finally, limitations in sequencing prevent us from identifying the exact bacterial species for most reads, therefore, the genera was the lowest level of classification. Additionally, the *Muribaculaceae* family had many unclassified genera, so it remained categorized at the family level. FSuture studies will also include an assessment of cell-specific markers to identify the contribution of the immune cell to the altered immune response. Additionally, males and females will be analysed separately to determine the sex-specific contributions to the gut and immune profile in this prepubescent period, also whether changes in bacteria and brain cytokines could also lead to behavioral change, however, here we were focused on specific changes in gut microbiome and immune response at 4–5 weeks regardless of sex. Despite the aforementioned limitations, our findings suggest that the administration of antibiotics during pregnancy and delivery results in alterations to the gut microbiota and immune profile of offspring and can cause long-lasting changes. 

In conclusion, maternal antibiotics during pregnancy results in modifications of gut microbiota in offspring mice in addition to the peripheral blood and the CNS immune response of offspring. These findings suggest that maternal antibiotics may be a risk factor for disrupted gut microbiota development in early life, resulting in long-term health deficits and leading to disease. This study highlights key gut microbiota and immune changes that occur during the post-weaning prepubescent stage can be altered by maternal ABX-treatments. Further investigation into the long-term effect of maternal ABX-treatment on offspring’s health should include more prepubescent mice.

## Supplementary Materials

The following supporting information can be downloaded at: https://www.mdpi.com/article/10.3390/ijms232112879/s1.
