# Supplementary material for: Prenatal Maternal Antibiotics Treatment Alters the Gut Microbiota and Immune Function of Post-Weaned Prepubescent Offspring
Source: Int J Mol Sci. 2022 Oct 25;23(21):12879. doi: 10.3390/ijms232112879 (PMC9655507; doi:10.3390/ijms232112879)
Supplement: Supplementary file 1 [file ijms-23-12879-s001.zip › ijms-1946176-supplementary.pdf]

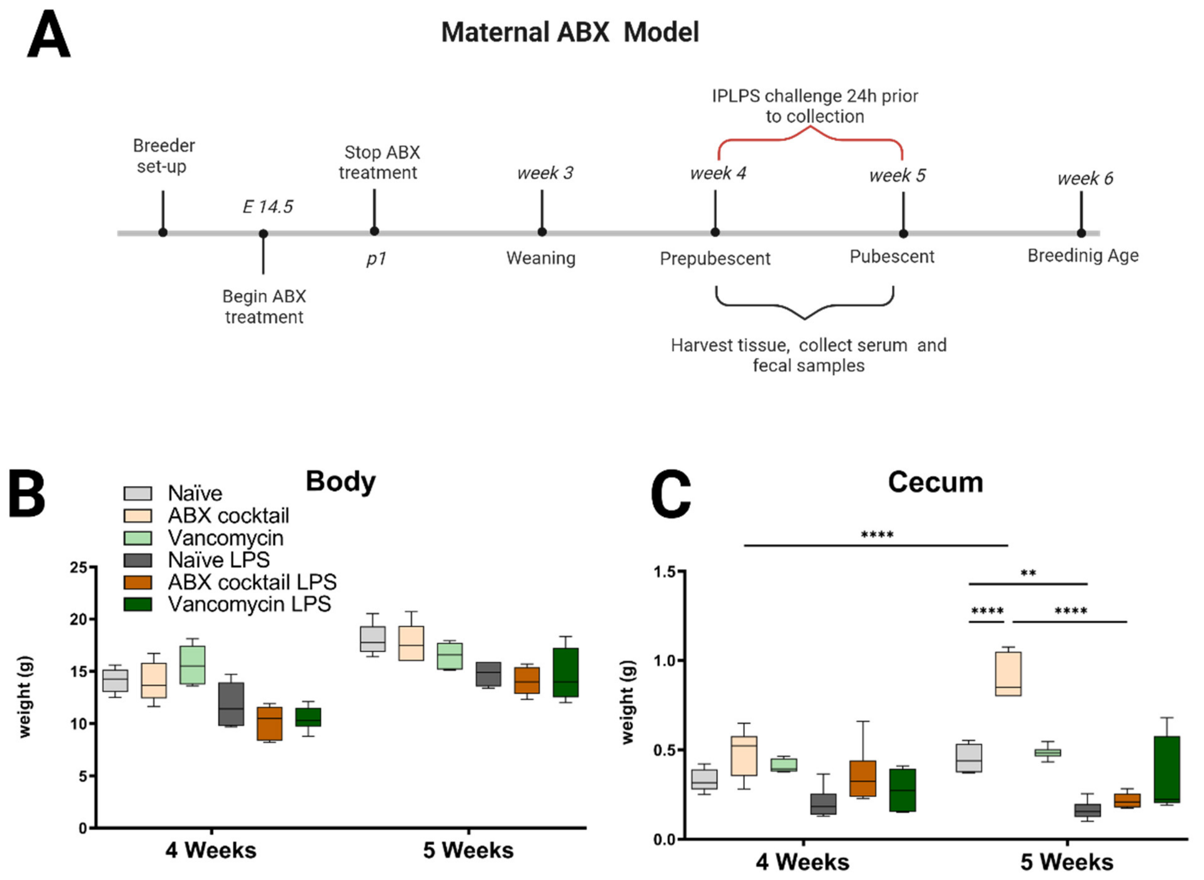

Figure S1. Prenatal ABX administration alters the gut microbiota diversity. (A, B) α-diversity measured using observed features and Shannon index (C) β-diversity indexes measured using principal coordinate analysis (PCoA) plot of Bray-Curtis dissimilarity between samples. Naïve = light grey, ABX cocktail = light brown, Vancomycin = light green, Naïve LPS = grey, ABX cocktail LPS = brown and Vancomycin LPS = green. N = 6 for all groups. Two-way ANOVA followed by Tukey post-correction test for multiple comparisons where values represent means ± SEM, *$p \leq 0.05$, **$p \leq 0.01$, ***$p \leq 0.001$ and ****$p \leq 0.0001$.

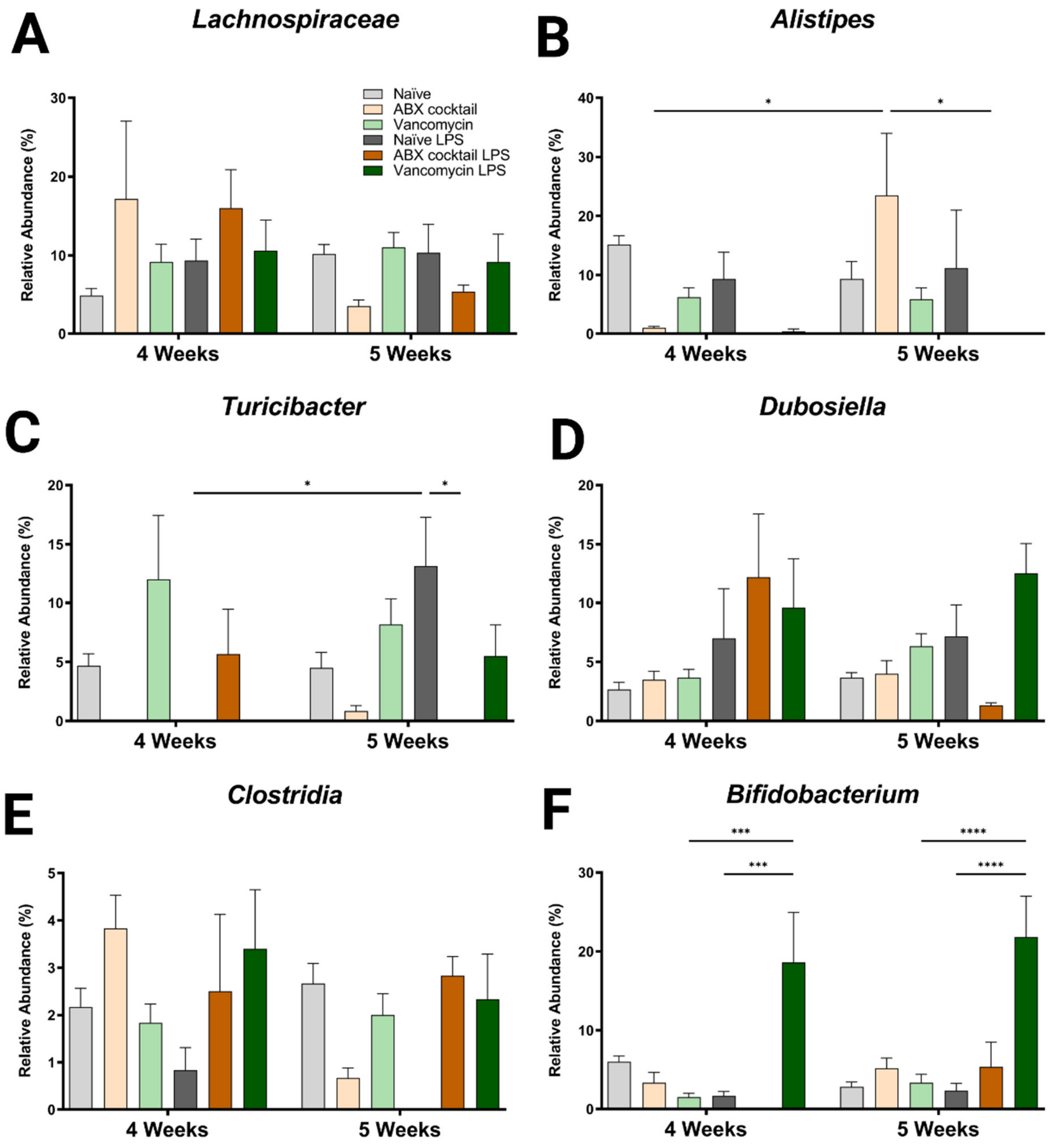

Figure S2. Maternal ABX alters additional offspring genus populations. Relative abundances of (A) *Lachnospiraceae*, (B) *Alistipes*, (C) *Turicibacter*, (D) *Dubosiella*, (E) *Clostridia*, and (F) *Bifidobacterium* at 4 and 5 weeks. Naïve = light grey, ABX cocktail = light brown, Vancomycin = light green, Naïve LPS = grey, ABX cocktail LPS = brown and Vancomycin LPS = green. N = 6 for all groups. Two-way ANOVA followed by Tukey post-correction test for multiple comparisons where values represent means ± SEM, *$p \leq 0.05$, **$p \leq 0.01$, ***$p \leq 0.001$ and ****$p \leq 0.0001$.

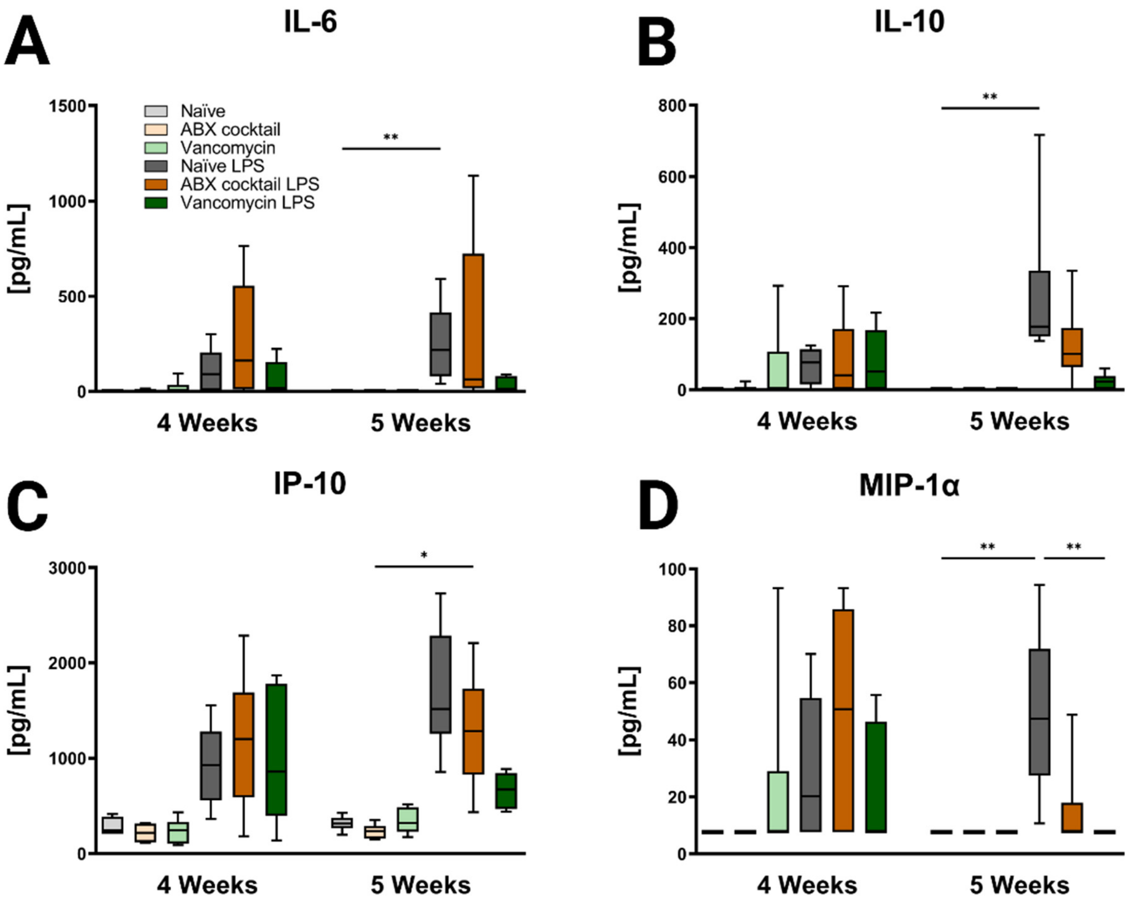

Figure S3. Additional inducible serum cytokines and chemokines affected by maternal ABX treatment. The data represents the responses of (A) IL-6, (B) MIP-2, a.k.a. CXCL2, (C) IP-10, and (D) MIP-1 α, a.k.a. CCL3 at 4 and 5 weeks. Naïve = light grey, ABX cocktail = light brown, Vancomycin = light green, Naïve LPS = grey, ABX cocktail LPS = brown and Vancomycin LPS = green. N = 6 for all groups. Kruskal-Wallis with Dunn's multiple comparison test. Values represent median + interquartile range means ± SEM, $^*p \leq 0.05$, $^{**}p \leq 0.01$, $^{***}p \leq 0.001$ and $^{****}p \leq 0.0001$.

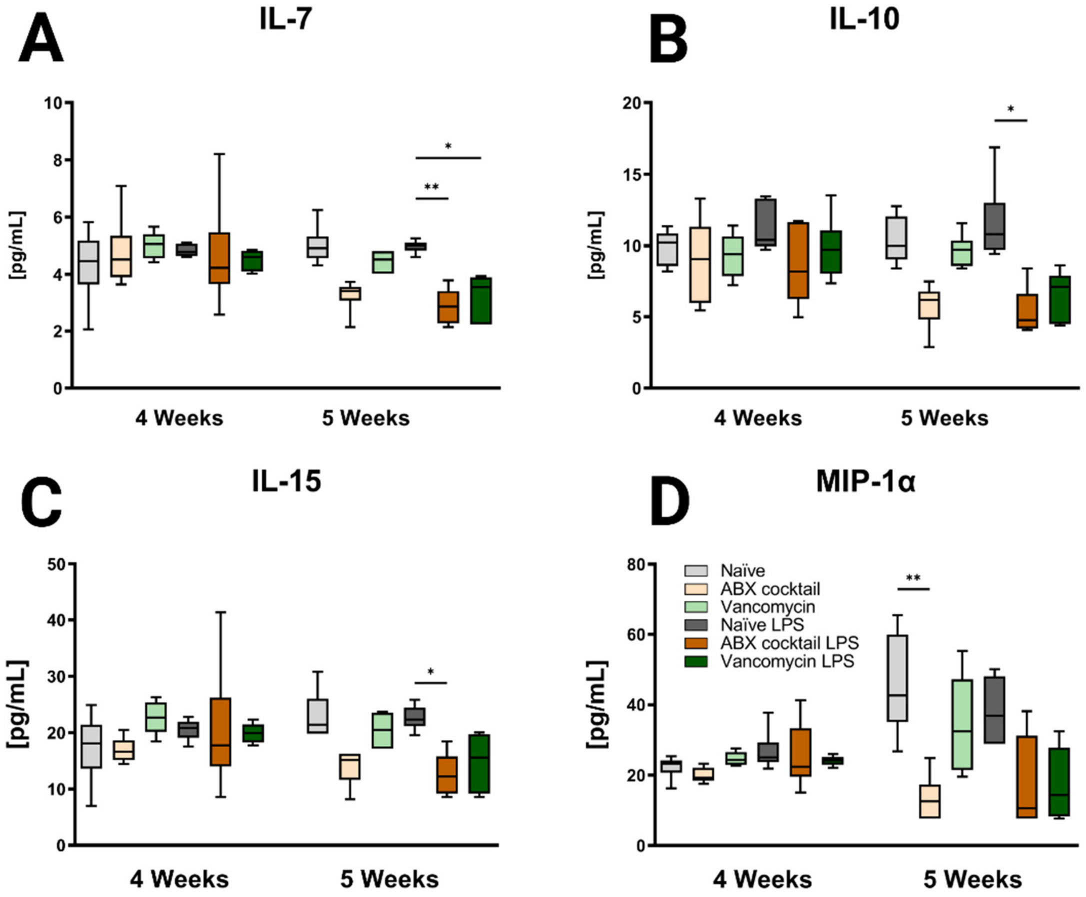

Figure S4. Additional inducible brain cytokines affected by maternal ABX treatment. Data showing the responses of (A) IL-7, (B) IL-10, (C) IL-15, and (D) MIP-1 α, a.k.a. CCL3 at 4 and 5 weeks. Naïve = light grey, ABX cocktail = light brown, Vancomycin = light green, Naïve LPS = grey, ABX cocktail LPS = brown and Vancomycin LPS = green. N = 6 for all groups. Kruskal-Wallis with Dunn's multiple comparison test. Values represent median + interquartile range means ± SEM, $*p \leq 0.05$, $**p \leq 0.01$, $***p \leq 0.001$ and $****p \leq 0.0001$.

Table S1. Percentage relative abundances of sequences classified to phylum at 4 weeks and 5 weeks, respectively, Individual phyla percentages compared to show differences between naïve and the ABX treatment groups.

		Percentage Relative Abundance in Samples		
		**Naïve n=6**	**ABX-cocktail n=6**	**Vancomycin n=6**
**Phylum**				
Actinobacteria	4 weeks	6	4	2
	5 weeks	4	5	4
Bacteroidetes	4 weeks	41	25	33
	5 weeks	38	39	32
Firmicutes	4 weeks	52	72	62
	5 weeks	58	55	61
Proteobacteria	4 weeks	1	0	3
	5 weeks	1	1	2
Total	4 weeks	100	101	100
	5 weeks	101	100	99
**Phylum post LPS**				
Actinobacteria	4 weeks	2	0	19
	5 weeks	3	6	22
Bacteroidetes	4 weeks	11	1	14
	5 weeks	24	47	1
Firmicutes	4 weeks	80	77	58
	5 weeks	67	41	70
Proteobacteria	4 weeks	6	21	6
	5 weeks	6	6	7
Total	4 weeks	99	99	97
	5 weeks	100	100	100
